# In Vitro and In Silico Biological Activities Investigation of Ethyl Acetate Extract of *Rubus ulmifolius* Schott Leaves Collected in Algeria

**DOI:** 10.3390/plants13233425

**Published:** 2024-12-06

**Authors:** Amina Bramki, Djamila Benouchenne, Maria Michela Salvatore, Ouided Benslama, Anna Andolfi, Noureddine Rahim, Mohamed Moussaoui, Sourore Ramoul, Sirine Nessah, Ghozlane Barboucha, Chawki Bensouici, Alessio Cimmino, Jesùs Garcìa Zorrilla, Marco Masi

**Affiliations:** 1 Laboratory of BioEngineering, Higher National School of Biotechnology Taoufik Khaznadar, Nouveau Pôle Universitaire Ali Mendjeli, BP. E66, Constantine 25100, Algeria; a.bramki@ensbiotech.edu.dz; 2 Higher National School of Biotechnology Taoufik Khaznadar, Nouveau Pôle Universitaire Ali Mendjeli, BP. E66, Constantine 25100, Algeria; d.benouchenne@ensbiotech.edu.dz (D.B.); s.ramoul98@gmail.com (S.R.); sirinsirin2000hh@gmail.com (S.N.); 3 Laboratory of Genetic, Biochemistry and Plants Biotechnology, Faculty of Natural and Life Sciences, University of Mentouri Brothers, Constantine 1, Constantine 25000, Algeria; 4 Department of Chemical Sciences, University of Naples Federico II, 80126 Naples, Italy; mariamichela.salvatore@unina.it (M.M.S.); andolfi@unina.it (A.A.); alessio.cimmino@unina.it (A.C.); 5 Laboratory of Natural Substances, Biomolecules, and Biotechnological Applications, Department of Natural and Life Sciences, Larbi Ben M’Hidi University, Oum El Bouaghi 04000, Algeria; ouided.benslama@univ-oeb.dz; 6 Biotechnologies Laboratory, Higher National School of Biotechnology Taoufik Khaznadar, Nouveau Pôle Universitaire Ali Mendjeli, BP. E66, Constantine 25100, Algeria; n.rahim@ensbiotech.edu.dz (N.R.); g.barb@ensbiotech.edu.dz (G.B.); 7 Pharmaceutical Sciences Research Center, Constantine 25100, Algeria; mohamed.moussaoui@univ-bejaia.dz; 8 Biotechnology Research Center, UV 03, BP. E73, Ali Mendjeli 25016, Algeria; c.bensouici@crbt.dz; 9 Allelopathy Group, Department of Organic Chemistry, Facultad de Ciencias, Institute of Biomolecules (INBIO), University of Cadiz, 11510 Puerto Real, Spain

**Keywords:** *Rubus ulmifolius*, GC-MS analysis, biological activities, molecular docking, ADMET properties

## Abstract

This investigation aimed to assess the in vitro and in silico biological properties of the ethyl acetate (EtOAc) extract obtained from leaves of *Rubus ulmifolius* Schott collected in Algeria. The phytochemical screening data disclosed that flavonoids, tannins, coumarins, saponins, and anthocyanins were abundant. High levels of total phenolics, total flavonoids and flavonols (523.25 ± 3.53 µg GAE/mg, 20.41 ± 1.80, and 9.62 ± 0.51 µg QE/mg respectively) were detected. Furthermore, GC-MS analysis was performed to identify low molecular weight compounds. d-(-)-Fructofuranose, gallic acid, caffeic acid, and catechin were detected as main metabolites of the EtOAc extract. The outcomes revealed that the extract exerted a potent antioxidant apt, and ensured significant bacterial growth inhibitory capacity, where the inhibition zone diameters ranged from 20.0 ± 0.5 to 24.5 ± 0.3 mm. These outcomes were confirmed through molecular docking against key bacterial enzymes that revealed significant interactions and binding affinities. d-(-)-Fructofuranose was identified as the most polar and flexible compound. Gallic acid and caffeic acid demonstrated higher unsaturation. Caffeic acid was well absorbed in the blood–brain barrier (BBB) and human intestine. Catechin was well absorbed in CaCO_3_, and can act as an inhibitor of CYP1A2. These results highlight how crucial it is to keep looking into natural substances in the quest for more potent and targeted pathology therapies.

## 1. Introduction

The geographical location of Algeria has led to a high diversity of plants, the majority of which are indigenous. However, not much is known or has been published about these aromatic and medicinal plants, which are abundant in secondary metabolites that have important pharmacological and therapeutic properties [1]. Historically, people have utilized various plant parts without realizing that these parts contained compounds that could heal a variety of diseases. In fact, the presence of bioactive compounds in natural essences can help treat a wide range of diseases, including cancer, diabetes, hypertension, and inflammation. Medicinal plants are a major focus of pharmacological research. In this framework, our attention was drawn to *R. ulmifolius* Schott, widely known as “Elm-leaved Bramble” [2], which is one of the plants that has attracted considerable interest due to its many traditional medicinal characteristics. This plant has a long history of usage in folk medicine to treat a number of skin disorders, including inflammation, itching, and eczema lesions [3]. It is also utilized as an antipyretic and carminative medication [4]. Furthermore, Lemus et al. [5] reported that *R. ulmifolius* was used in traditional Chinese medicine for its hypoglycemic properties. Its leaves and young shoots have also been utilized for their beneficial properties, which include their anti-inflammatory, antimicrobial, and spasmolytic effects on the gastrointestinal tract. In this regard, Tabarki et al. [6] proved that the leaves have a potent antioxidant effect and significant antimicrobial capacity against a wide range of bacteria. In addition, the fruits are considered healthy foods since they help prevent metabolic syndrome on multiple levels, which include heart disease and type II diabetes [7]. The growing interest in *R. ulmifolius* stems from its abundance of several phytochemical components, such as flavonoids, tannins, anthocyanins, and phenolic acids, which are well-known for their numerous biological activities [8]. The integration of the phytochemical profile and biological activities of this essence offers a holistic strategy for optimizing the medicinal benefits of this plant, along with encouraging opportunities for the development of novel natural therapeutic agents. The current work used GC-MS analysis to examine the chemical constituents of the ethyl acetate (EtOAc) extract from *R. ulmifolius* Schott. This was undertaken in order to evaluate its biological activities, such as its ability to fight various bacterial strains both in vitro and in silico (using molecular docking) and their antioxidant activity in vitro via several assays with various mechanisms, including DPPH^•^, ABTS^•+^, phenanthroline, and FRAP. The modulation of the gene expression profile as well as the ADMET features (absorption, distribution, metabolism, excretion, and toxicity) were also investigated. This paper provides the first comprehensive description of the bioactive properties in vitro and in silico of *R. ulmifolius* and emphasizes the plant’s potential as a natural source of antioxidants and antibacterial agents.

## 2. Results and Discussion

In order to better understand the chemical profile or secondary compounds found in the leaves of the plant *R. ulmifolius* Schott, a maceration extraction was carried out, followed by fractionation using three different solvents with increased polarities to aid in quantification and enhance secondary compound purity. The EtOAc extract was chosen for investigation due to its ability to concentrate relatively polar constituents, such as flavonoids, phenolic acids, tannins, sterols, and terpenoids, which are well-known for their diverse biological properties [9]. This selection was based on the potential of these compounds to contribute to significant pharmacological and therapeutical activities. The extraction of bioactive molecules using ethyl acetate solvent yielded a relatively high yield (4.4%), falling within the range of results established by Ali et al. [8], which revealed extraction yields of *R. ulmifolius* Schott leaves using various solvents ranging from 4.38% to 15%. Indeed, the extraction yield may vary depending on several factors, including the extraction techniques used, the plant’s growth conditions, the harvesting period, and genetic characteristics [10,11].

### 2.1. Phytochemical Screening

The phytochemical screening tests were conducted using tubes in order to check and draw the major classes of secondary metabolites found in plants. The outcomes of the phytochemical screening of the EtOAc extract from *R. ulmifolius* leaves are presented in Table 1.

The findings of the phytochemical screening indicated that the extract from this essence was rich in tannins, followed by flavonoids, coumarins, saponins, and anthocyanins. Our results are in line with those of Ali et al. [8], who worked on the leaves, and Akhtar et al. [12], who worked on the plant’s aerial part. This demonstrated the plant *R. ulmifolius*’s abundance in secondary metabolites, which endows it with remarkable pharmacological properties. The study by Martins et al. [13] listed the various therapeutic indications that it may have as well as the reasons for traditional medicine using it.

### 2.2. Determination of Total Phenolic, Total Flavonoid, and Flavonol Contents

Table 2 presents the results of the total phenolics, flavonoids, and flavonols in the plant *R. ulmifolius*’s EtOAc extract.

The findings showed that the EtOAc extract had a very high concentration of polyphenols, flavonoids, and flavonols, with values of 523.25 ± 3.53 µg GAE/mg, 20.41 ± 1.80, 9.62 ± 0.51 µg QE/mg of extract, respectively (Table 2). To the best of our knowledge, no research has been done to ascertain the amounts of polyphenols and flavonoids found in the leaves of the species *R. ulmifolius*, nor has there been any report published regarding the concentration of flavonols in either the genus or the plant under investigation. The polyphenol outcomes are higher than those of Akkari et al. [14], who examined extracts from *R. ulmifolius* fruits (distilled water, n hexane, chloroform, and methanol) and stated that the polyphenol content ranged from 1.57 ± 0.10 to 64.54 ± 0.94 mg GAE/g DW, while the flavonoid content was found to be between 0.71 ± 0.03 and 28.06 ± 0.19 mg QE/g DW. Additionally, Desmiaty et al. [15] examined methanolic extracts of leaves belonging to the same genus and disclosed that *R. fraxinifolius* had a polyphenol content of 39.0 ± 26.5 mg GAE/g of extract while *R. rosifolius* had a content of 80.62 ± 21.6 mg GAE/g of extract. Similarly, Yousefbeyk et al. [16] estimated that the polyphenol contents of ethyl acetate fractions from the leaves and roots of *R. hyrcanus* Juz were 82.54 ± 0.23 and 292.32 ± 0.02 mg GAE/g of extract, respectively, while the flavonoid contents were 47.11 ± 0.04, and 7.38 ± 0.02 mg QE/g extract. The disparity in flavonoid and phenolic levels between studies can be attributed to a variety of factors, including plant origin, ambient conditions, phenological stage, extraction methods, and the polarity of the organic solvents used [17].

### 2.3. GC-MS Analysis

**Figure 1:**
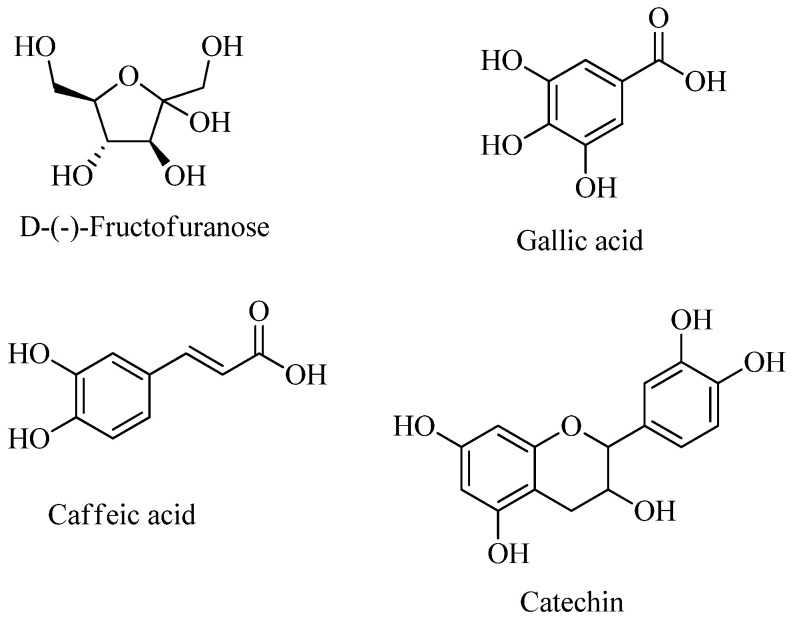
Chemical structures of compounds identified in the crude EtOAc extract by GC-MS.

The crude EtOAc extract obtained from *R. ulmifolius* leaves has been analyzed by GC-MS as described in the Section 3.6 to detect the main low molecular weight compounds. Table 3 and Figure 1 show the four main metabolites identified. Figure S1 provides the annotated total ion current chromatogram (TICC) from GC-MS analysis of the crude EtOAc extract.

### 2.4. Antioxidant Ability

The evaluation of antioxidant activity using four different methods demonstrated the significant antioxidant effect of the EtOAc fraction from the selected plant. The results summarized in Table 4 provide the inhibition concentration expressed as IC_50_ (µg/mL) for DPPH^•^ and ABTS^•+^, and A_0.5_ (µg/mL) values for phenanthroline and FRAP.

The DPPH^•^ free radical scavenging capacity results showed that the extract had a moderate significant activity with an IC_50_ = 98.82 ± 1.01 μg/mL, which can be compared with ascorbic acid (IC_50_ = 4.40 ± 0.10 μg/mL) and Trolox (IC_50_ = 5.14 ± 0.09 μg/mL).

Regarding antioxidant activity against the ABTS^•+^ cation radical, the data obtained indicated that the EtOAc extract had a significant activity, with an IC_50_ value of 4.20 ± 0.13 μg/mL, which is near to the results obtained using the Trolox (IC_50_ = 3.27 ± 0.17 μg/mL) and ascorbic acid (IC_50_ = 3.07 ± 0.05 μg/mL) standards. On the other hand, in metal chelation tests, the extract demonstrated a moderate inhibitory activity (A_0.5_ = 21.22 ± 0.59 μg/mL) in the reduction of Fe^2+^-phenanthroline complex, as compared to Trolox (A_0.5_ = 5.21 ± 0.07 μg/mL) and ascorbic acid (A_0.5_ = 3.08 ± 0.05 μg/mL). Furthermore, the FRAP test findings showed that the investigated extract had a reasonably considerable iron reduction capacity (A_0.5_ = 94.09 ± 1.40 μg/mL) when compared to the two ascorbic acid (A_0.5_ = 3.76 ± 0.23 μg/mL) and Trolox (A_0.5_ = 5.43 ± 0.31 μg/mL) standards examined. Indeed, numerous pieces of research on the *Rubus* species have in fact shown that this group contains chemicals with antioxidant capabilities, which have positive effects on world health [18]. According to Mawazi et al. [19], the antioxidant and sun protection properties of the bioactive ingredients found in some *Rubus* species boost the use of this plant in cosmetic products. Also, the results of Ruiz-Rodríguez et al. [20] and Schulz et al. [21] demonstrated that *R. ulmifolius* fruits have a high value in bioactive components and a high antioxidative capacity. Additionally, Ali et al. [8] studied the antioxidant apt of the aerial parts of *R. ulmifolius* and showed that the crude extract’s quenching of free radicals’ activity in DPPH^•^ assay is comparable to that of ascorbic acid. Likewise, Martini et al. [22] examined the antioxidant ability of *R. ulmifolius* leaves using the ABTS^•+^ test, and the data revealed a powerful capacity for scavenging ABTS^•+^ free radicals in comparison to Trolox. The GC-MS analyses findings revealed the presence of catechin, gallic acid, and caffeine compounds. In fact, these compounds’ antioxidant activity is well documented; in the same study [22], caffeic acid and gallic acid isolated from *R. ulmifolius* leaves showed stronger antioxidant activity than Trolox. Badhani et al. [23] reported that gallic acid and its derivatives have a potent antioxidant ability. Furthermore, Espíndola et al.’s [24] research indicated that caffeic acid and its derivatives have potential antioxidant, anti-inflammatory, and anti-carcinogenic properties. Its potent antioxidant action inhibits Reactive OS generation, hence lowering oxidative stress. Moreover, Bernatoniene and Kopustinskiene [25] reported that catechins possess a potent antioxidant capacity, which might be due to the structure of the molecules as well as the number and location of hydroxyl groups or their substituents. These molecules play a crucial role in eliminating lipid peroxidation products. Additionally, they may have an indirect antioxidant role by stimulating the endogenous antioxidant system, which controls enzymatic activity and signaling pathways [26].

### 2.5. Antibacterial Activity

Moreover, to free radicals and their harmful effects, bacterial strains resistance to many antibiotics is another problem for the human body, which caused the increasing in bacterial infections [27], all these stimulate and oblige the researchers to look for therapeutic alternatives from natural origins. Table 5 represents the zone inhibitions in mm exhibited by the EtOAc extract against the tested bacterial strains using the well diffusion technique.

The results showed that the EtOAc extract had a significant antibacterial effect against all the strains examined with inhibition diameters ranging from 20 ± 0.5 mm to 24.5 ± 0.3 mm, considerably greater than gentamicin positive control with diameters from 10.0 ± 0.2 mm to 19.1 ± 0.8 mm. Our findings are in agreement with those of Panizzi et al. [28], who proved that extracts of the plant *R. ulmifolius* have an antibacterial effect against *S. aureus*, *B. cereus*, *P. aeruginosa*, and *E. coli*, with inhibition zones ranging from 8.1 to 25 mm in diameters. In the same framework, it has been confirmed in a study by Martini et al. [22] that the extract of the leaves of *R. ulmifolius* display a significant antibacterial effect against *Helicobacter pylori* strains. Also, Quave et al. [29] reported that an extract from *R. ulmifolius* root inhibited *S. aureus* biofilm formation. Similarly, the findings of Ibba et al. [30] revealed that different extracts from *R. ulmifolius* leaves exhibited a potential effect on the *Streptococcus mutans* bacterial strain. With regard to MIC, the outcomes of this extract appeared to be effective even at low concentrations, with values ranging from 0.78 mg/mL for *B. subtilis*, *S. aureus*, *E. coli*, and *S. typhimurium*, to 3.12 mg/mL for *K. pneumoniae*. These results are superior to those obtained by Da Silva et al. [31], who demonstrated that the fruit extract of *R. ulmifolius* displayed a potent growth inhibitory apt against all tested strains (Gram+ and Gram-), with CMI values ranging from 2.5 to 20 mg/mL. In the same context, Yousefbeyk et al. [16] reported that extracts from the leaves and seeds of *R. hyrcanus* Juz. exhibited CMI values ranging from 1.5 to 100 mg/mL. The chemical profile of EtOAc from the selected plant using GC-MS analysis revealed the presence of catechin, gallic acid, d-(-)-fructofuranose, and caffeic acid. These components might be responsible for the potential of this extract’s antibacterial effect. According to studies by Passos et al. [32] and Tian et al. [33], gallic acid has a strong antimicrobial activity and can prevent the formation of biofilms. Furthermore, studies conducted by Kępa et al. [34] have demonstrated that caffeic acid presented varying effects on the *S. aureus* bacterial strain, with CMI values ranging from 256 μg/mL to 1024 μg/mL. It has been demonstrated by Khan et al. [35] that caffeic acid has been widely used to combat chronic infections caused by microbes such as bacteria, fungi, and viruses. Also, catechins have a potential bactericidal effect that is promising against Gram+ and Gram- bacteria, including the resistance of medication-resistant bacteria. Furthermore, these molecules inhibit the activity of virulence factors, specifically toxins, hence decreasing the pathogenicity of some bacteria [36].

### 2.6. In Silico Study

#### 2.6.1. Molecular Docking

**Figure 2:**
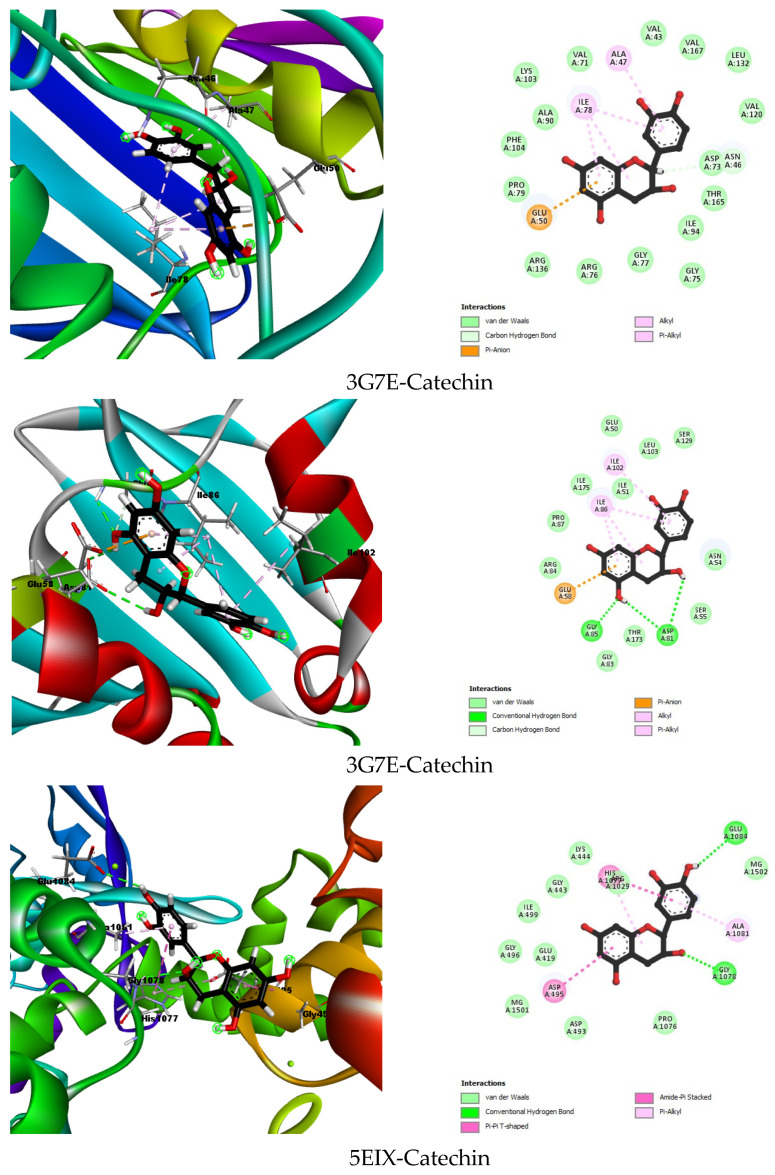

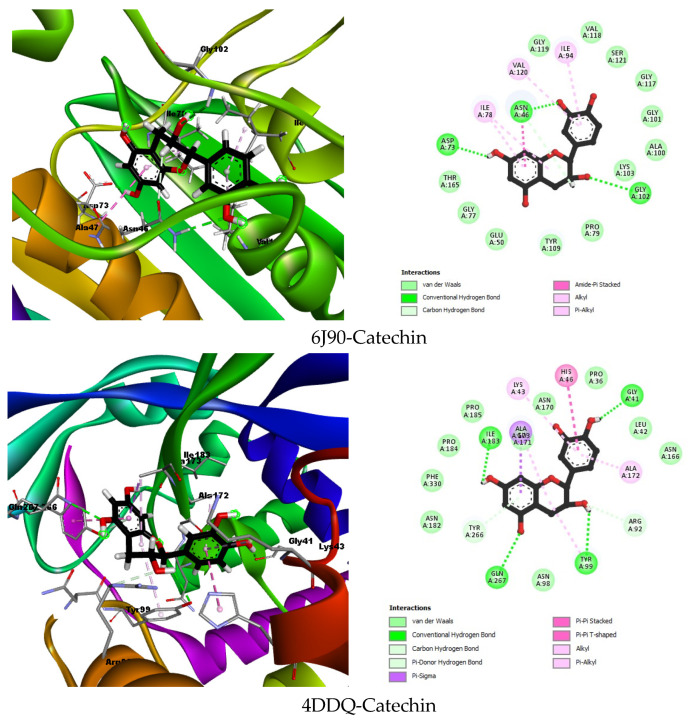
2D and 3D visualizations of the best-docked compound interaction within the active site for each studied enzyme.

Table 6 and Figure 2 summarizes the docking results of the reference molecules and the best-docked compounds for each studied enzyme. The binding energy, hydrogen interactions, and hydrophobic interactions were analyzed to determine the effectiveness of the plant compounds.

The enzymes selected for in silico analysis play crucial roles in bacterial survival and proliferation, making them prime targets for antibacterial agents. DNA gyrase and topoisomerase IV are essential for bacterial DNA replication, transcription, and cell division. Inhibiting these enzymes disrupts DNA processes, leading to bacterial cell death [37,38]. The chosen enzymes, 3G75 (DNA gyrase of *S. aureus*), 3G7E (DNA gyrase of *E. coli*), 4DDQ (DNA gyrase of *B. subtilis*), 5EIX (topoisomerase of *K. pneumoniae*), and 6J90 (DNA gyrase of *S. typhimurium*), are critical for antibacterial activity against the corresponding bacteria.

The molecular docking analysis provided valuable insights into the binding interactions and potential efficacy of plant extract compounds against key bacterial enzymes. The primary focus was on evaluating the antibacterial activity of these compounds through their binding affinities and interactions with the chosen enzymes. The results for the best-docked compound, catechin, and the co-crystallized ligands for each enzyme are summarized in Table 1.

The co-crystallized ligand B46 demonstrated a binding energy of −10.7579 kcal/mol with several hydrogen interactions, including Asp73, Asn56, Arg136, Gly102, Gly77, Arg76, and Asp49, and extensive hydrophobic interactions with residues such as Val43, Val120, Leu130, Val167, Met95, Ile94, His116, Ala47, Ile78, and Pro79. In contrast, catechin exhibited a significantly lower binding energy of −5.6915 kcal/mol. Catechin formed hydrogen interactions with Asn46 and had hydrophobic interactions with Ala47 and Ile78. Although the binding energy of catechin was lower than that of B46, it still formed critical interactions, suggesting potential antibacterial activity, albeit less potent than the co-crystallized ligand.

The co-crystallized ligand B48 showed a binding energy of −6.1928 kcal/mol with hydrogen bonds involving Asp81 and Ser55, and hydrophobic interactions with Ile175, Val79, Ile51, Ile86, Gly85, and Asn54. Catechin, as the best-docked compound, had a comparable binding energy of −6.1220 kcal/mol. Catechin formed hydrogen bonds with Gly85 and Asp81 and hydrophobic interactions with Ala102 and Ile86. The close binding energy and similar interaction profile of catechin to the co-crystallized ligand suggest that catechin could be an effective antibacterial agent against *S. aureus*.

The co-crystallized ligand LFX had a binding energy of −6.3059 kcal/mol, forming hydrogen bonds with Ser1080, Lys444, and Lys442. Catechin, with a binding energy of −3.6767 kcal/mol, interacted through hydrogen bonds with Glu1084 and Gly1078 and exhibited hydrophobic interactions with Asp495, Ala1081, and His1077. Although catechin’s binding energy was lower, its specific interactions indicate a potential, albeit weaker, antibacterial effect against *K. pneumoniae*.

The co-crystallized ligand ATP had a binding energy of −10.3302 kcal/mol, forming hydrogen bonds with Asp73, Gly102, Ser121, Val118, His116, Leu115, Gly119, Gly117, Val120, and Asn46, and a hydrophobic interaction with Ile78. Catechin, showing a binding energy of −7.0767 kcal/mol, formed hydrogen bonds with Ans46, Asp73, and Gly102, and hydrophobic interactions with Ile78, Ile94, and Val120. Despite a lower binding energy, catechin’s significant interactions with the active site residues suggest it might have considerable antibacterial potential against *S. typhimurium*.

Catechin, as the best-docked compound for 4DDQ, demonstrated a binding energy of −6.3718 kcal/mol. It formed hydrogen bonds with Ile183, Gln267, Tyr99, Arg92, and Gly41, and hydrophobic interactions with Ala171, Lys43, His46, and Ala172. The interactions with crucial active site residues and a relatively low binding energy indicate catechin’s potential efficacy against *B. subtilis*.

Although catechin displayed a lower binding energy compared to the co-crystallized ligands, it remains a promising candidate due to its natural origin, which often correlates with reduced toxicity, and its potential for multi-target interactions. These properties may complement its binding affinity and make it a valuable lead compound for further optimization. Future work could explore structural modifications or synergistic effects with other compounds to enhance its inhibitory activity.

Catechin has been extensively studied for its antibacterial properties, including its ability to inhibit quorum sensing (QS) in pathogens like *P. aeruginosa*. Docking studies have demonstrated its interaction with key QS regulators, such as LasR, through hydrogen bonds with specific residues, impacting biofilm formation and virulence [39]. These findings align with our results, reinforcing the potential of catechin as a bioactive compound targeting bacterial systems.

Furthermore, Kurnia et al. highlighted catechin’s inhibitory effects on bacterial systems, particularly its potential to disrupt quorum sensing or inhibit bacterial efflux pumps through binding at specific protein sites [40].

Additionally, previous studies investigating catechin’s molecular interactions with bacterial enzymes and receptors corroborate its significant binding potential. For instance, catechin has been found to target bacterial DNA Gyrase and ATP-binding proteins, underlining its role in bacterial growth inhibition [41]. By contextualizing our results within this body of work, our study further validates catechin’s therapeutic potential, particularly when combined with other agents targeting critical bacterial systems.

#### 2.6.2. ADMET Properties

(a) Canonical SMILES determination

The canonical SMILES, chemical structure, and molecular formula were identified using the Pubchem database.

(b) Physicochemical properties of molecules

The physicochemical properties of the identified compounds are reported in Table S1 and Figure S2.

Dr. Lipinski created five fundamental parameters to evaluate a compound’s appropriateness for pharmaceutical application [42,43,44]. Lipinski’s Rule of Five states that molecules that satisfy the following requirements are the best candidates for drug development: they should have a molecular weight of no more than 500 Daltons, a maximum of five hydrogen bond donors, and a maximum of ten hydrogen bond acceptors. Furthermore, substances with high bioavailability and a log P coefficient just above 5 are seen to be promising drug-like candidates.

Table S1 demonstrates that every molecule under investigation falls inside the permissible range, according to Lipinski’s Rule of Five, of 170.12 to 340.12 Daltons for molecular weight. Furthermore, all of the examined substances have log *p* values that are less than 5, which indicates hydrophilicity. In particular, d-(-)-fructofuranose exhibits excellent solubility because of its hydrophilic character, which is shown in its extremely low log *p* value. Table S1 shows that all of the compounds have less than 10 hydrogen bond acceptors, except d-(-)-fructofuranose. The latter is distinguished by having more hydrogen bond donors than other molecules. Size, lipophilicity (LIPO), insolubility (INSOLU), unsaturation (INSATU), flexibility (FLEX), and polarity (POLAR) were the six factors evaluated by the Swiss ADME web server.

- Polarity: d-(-)-fructofuranose was found to be the most polar.
- Unsaturation: The high degree of unsaturation was present in gallic acid and caffeic acid followed by catechin and d-(-)-fructofuranose, respectively.
- Flexibility: d-(-)-fructofuranose showed the most flexibility.
- Lipophilicity and insolubility: The outcomes of all the examined compounds were comparable in these categories.

(c) Pharmacokinetic properties

Drug discovery and the evaluation of chemical safety depend heavily on the features of ADMET (absorption, distribution, metabolism, excretion, and toxicity). Drug development failure is mostly caused by undesirable pharmacokinetic features and unacceptable toxicity, which represent potential dangers to human health and the environment [45].

It is becoming increasingly possible to anticipate the pharmacokinetic features of chemical substances with accuracy and timeliness [46]. The evaluation of these chemicals using traditional procedures takes a lot of time and labor [47]. A thorough understanding of the pharmacological mechanisms of medicinal plants is necessary to modernize their application. Thanks to developments in computer technology, the emergence of in silico methods has completely changed the prediction of chemical-biological target interactions.

Table S2 represents the ADMET properties examined via the ADMET sar3 server. Absorption is how drugs are transported into the human circulatory system. The outcomes showed that caffeic acid was well absorbed by the blood–brain barrier (BBB 72.3%) (Figure S3), and the human intestine (83.5%), and these findings corroborate those reported by Olthof et al. [48] using an in vivo experiment, while catechin was well absorbed in CaCO_2_. Results of 20% bioavailability (F20) and 30% bioavailability (F30) indicate that caffeic acid presented high bioavailability, with values of 59.6% and 71.7%, respectively. Distribution is the process by which substances cross the cell membrane to enter different cells. All molecules can be inhibitors of OATP1B1 and OATP1B3, with percentages ranging from 82.7 to 90.5% and 81.1 to 90.5%, with a score of 1. In the same context, catechin displayed a strong interaction with Plasma Protein Binding (PPB), followed by gallic acid and caffeic acid. No molecules can be either a substrate or Pgp inhibitor. Once metabolism occurs, the original compound is transformed into new compounds called metabolites. The molecule drug metabolism is assessed by cytochrome P450 enzymes in the liver. Metabolically, catechin can act as an inhibitor of CYP1A2, followed by gallic acid. In the same regard, all compounds can be a substrate of UGT, with values of 97.5%, 96.4%, 95%, and 75.7%, respectively. The process by which a drug’s original form and its metabolites are eliminated from the body is known as excretion. The outcomes showed that catechin was well eliminated by plasma clearance (CLp), followed by gallic acid and caffeic acid, while all molecules can be eliminated renally (CLr). The organ’s toxicity, d-(-)-fructofuranose, gallic acid, and caffeic acid can cause neurotoxicity. d-(-)-Fructofuranose and catechin can cause respiratory toxicity. Gallic acid and caffeic acid can be a cause of eye irritation and skin corrosion, while d-(-)-fructofuranose and gallic acid can cause acute dermal toxicity. All the examined compounds can lead to hemolytic toxicity. The potential hemolytic activity of the four compounds identified in the plant extract represents a significant limitation to their direct therapeutic application. However, this finding serves as a crucial step in guiding future research directions. Structural modifications to reduce hemolytic toxicity, while preserving antibacterial potency, should be a priority. Likewise, gallic acid and catechin can be the cause of mitochondrial toxicity. From the obtained results, all molecules complied with Lipinski, Pfizer, and GSK rules; as a result, they are acceptable to be used as drug-promoting molecules.

(d) Cytotoxicity of the compound’s prediction

Numerous bioactive substances, such as those with anti-cancer, anti-diabetic, and anti-Alzheimer’s impacts, are produced by plants and have the potential to be used therapeutically. The CLC-Pred server was used to assess these drugs’ cytotoxicity against tumor cell lines. The cytotoxic effects on cancer cells of the chemicalsare compiled in Table 7.

To the best of our knowledge, no reports about the cytotoxic effect of d-(-)-fructofuranose have been published. The results showed that caffeic acid was an effective adenocarcinoma cancer inhibitor, with a noteworthy cytotoxic capacity against ovarian adenocarcinoma (IGROV-1 cell line), with an activity ratio of 0.607 compared to 0.010. Gallic acid may have inhibited the growth of the Hs 683 cell line in glioma brain cancer.

The literature has extensively documented the anti-cancer benefits of caffeic acid. According to Kleczka et al. [49], caffeic acid phenethyl ester at specific doses exhibited cytotoxic effects on ovarian cancer cells. This compound was also found to have potent antiproliferative, cytotoxic, and pro-apoptotic properties against cancer cells of the head and neck, as well as against breast, colorectal, and lung cancers, and leukemia. Furthermore, Yang et al. [50] demonstrated that the Temozolomide/gallic acid (TMZ/GA) combination increased the suppression of cellular viability and apoptotic level in the U87MG glioma cell line when compared to the actions of TMZ alone or GA alone. In the same framework, the outcomes of Paolini et al. [51] revealed that miRNA expression varied with GA doses and validated in T98G cells the anti-proliferative action of GA reported for other glioma cell lines. Furthermore, Jafarinejad et al. [52] found that treating A549 cells with different dosages of gallic acid (20, 30, and 40 μM) significantly reduced cell survival after 12, 24, and 48 h. In the same framework, Varela-Rodríguez et al. [53] found that treating SKOV-3 cells with gallic acid caused cytotoxic activity by producing ROS, altering the cytoskeleton and inducing apoptosis. Moghtaderi [54] found that the combination of gallic acid and curcumin significantly reduced MDA-MB-231 cell proliferation. Catechin is a polyphenol whose biological activities have been widely reported, including antioxidant, antibacterial, and anti-cancer agents. In this regard, Meneses-Gutiérrez et al. [55] displayed that the oligomers of catechin, epicatechin, and resveratrol had a significant anti-viability effect on the T24 cell line of human urinary bladder transitional cell carcinoma. These findings underscore caffeic acid and gallic acid’s potential as therapeutic agents in the treatment of glioma and potentially other types of cancer.

## 3. Materials and Methods

### 3.1. Collection and Identification of the Plant

*R. ulmifolius* Schott leaves were collected in September 2023 from Jijel, in the province of Bab Sour (36°49′12.2″ N 5°45′45.9″ E). Authentication of the plant was carried out by Dr. Moussaoui Mohamed (Botanist Researcher at Pharmaceutical Sciences Research Center, Constantine). The samples were dried in a dry place and powdered using a grinder, after which they were stored for further analyses.

### 3.2. Extraction of Secondary Metabolites

The extraction of secondary metabolites was realized using a modified protocol of Zatout and Kacem Chaouche [56]. The extraction process was carried out by cold maceration, which involved soaking a quantity of 20 g of powdered leaves in 300 mL of methanol: H_2_O (1:1) and shaking the mixture for 24 h at room temperature. After filtration, the solvent was then removed under reduced pressure by rotary evaporator apparatus at 30 °C. The aqueous phase was extracted with *n*-hexane (3 × 300 mL) then dichloromethane (3 × 300 mL) and finally with ethyl acetate (3 × 300 mL). The latter fraction was taken for further analyses.

### 3.3. The Yield of Extraction

The extraction yield (Y) of the extract was defined as the ratio between the weight of extract obtained (g) and the weight of the dried powdered plant material (g), as reported by Yakoubi et al. [57]. This yield was calculated using the following formula:

$$Y(\%)=\frac{weight\;of\;extract\;collected}{weight\;of\;the\;plant\;material}\times 100$$

### 3.4. Phytochemical Screening

The phytochemical screening was carried out according to the method described by Benouchenne et al. [58].

- Test for Flavonoids

A volume of 2 mL of the EtOAc extract was mixed with 0.5 mL of concentrated hydrochloric acid and 0.5 g of metal magnesium. A pink/red coloration developing after 3 min indicated the presence of flavonoids.

- Test for Tannins

A few drops of 1% ferric chloride were added to 1 mL of the extract. The appearance of a blue/dark color revealed the presence of gallic tannins; however, a green/blue developed color proved the existence of catechin tannins.

- Test for Coumarins

In a test tube, a few drops of distilled water were added to 1 mL of the extract. The tube was covered with sodium hydroxide (10%)-soaked paper, and after that the tube was heated till ebullition. The appearance of yellowish fluorescence under a UV lamp (366 nm) indicated the presence of coumarins.

- Test for Alkaloids

A total of 1 mL of diluted sulfuric acid (50%) was added to 5 mL of the extract. After that, two tubes were prepared. One contained 2 mL of the acidified extract and 1 mL of Mayer’s reagent. The second tube had 2 mL of the extract and 1 mL of Dragendorff–Wagner’s reagent. The appearance of turbidity/precipitation indicated the presence of alkaloids.

- Test for Triterpenes, Sterols and Steroids

A volume of 5 mL of the extract was evaporated. The dried extract was dissolved in a mixture of acetic anhydride: chloroform (5:5) (*v*/*v*). After that, a few drops of concentrated sulfuric acid were added. The appearance of green coloration indicated the presence of steroids, while the appearance of purple coloration revealed the presence of triterpenes.

- Test for Saponins

A total of 9 mL of distilled water was added to 1 mL of the extract which had been placed in a test tube and shaken vigorously for 15 s, and then the extract was left to stand for 10 min. The formation of stable foam (1 cm) indicated the presence of saponins.

- Test for Reducing Sugars

A total of 1 mL of distilled water was added to a test tube containing 1 mL of the extract. The mixture was heated till ebullition. After this, twenty drops of Fehling’s solution were added. The observed brick-red precipitate disclosed the presence of reducing sugars.

- Test for Anthocyanins

A volume of 2 mL of the extract, 5 mL of concentrated sulfuric acid, and 5 mL of ammonium hydroxide were added. The appearance of pink/red or blue coloration revealed the presence of anthocyanins.

### 3.5. Determination of Total Phenolics, Total Flavonoids, and Flavonols

#### 3.5.1. Determination of Total Phenolic Content (TPC)

The total phenolic content (TPC) in the EtOAc fraction was determined using the Folin–Ciocalteu Reagent (FCR) method as described by Singleton and Rossi [59]. A volume of 20 µL of plant extract was combined with a volume of 80 µL of sodium carbonate (7.5% *w*/*v*) and 100 µL of FCR (diluted 1:10 with deionized water). Two hours were spent incubating the reaction mixture in darkness. A microplate reader was used to determine the color’s absorbance at 765 nm. Gallic acid was used as a standard to produce a standard curve (Y = 0.0041X + 0.207; R^2^ = 0.9738), from which the TPC was derived. The results were expressed as µg of gallic acid equivalency per mg of dry extract.

#### 3.5.2. Determination of Total Flavonoid Content

With a few minor adjustments, the aluminum nitrate colorimetric assay from Topçu et al. [60] was used to determine the total flavonoid concentration (TFC) in the EtOAc extract. A volume of 50 µL of the sample at different concentrations was mixed with 10 µL of potassium acetate (CH_3_COOK; 9.8% *w/v*), and 10 µL of aluminum nitrate (Al(NO_3_)_3_, 9H_2_O). The mixture was incubated for 40 min. At 415 nm, the absorbance was measured. To prepare the blank, the extract was substituted with methanol. The quercetin calibration curve (Y = 0.0085X + 0.0471; R^2^ = 0.9954) was utilized to determine the total flavonoid concentration, which was then represented as µg of quercetin equivalency per mg of dry extract.

#### 3.5.3. Determination of Total Flavonol Content

The total flavonol content was measured using the protocol of Kumaran and Joel Karunakaran [61]. A volume of 50 µL of plant extract at different dilutions was mixed with 50 µL of aluminium trichloride (AlCl_3_, 2%) and 150 µL sodium acetate (5%). The mixture was incubated in the dark for 90 min. The absorbances were measured at 440 nm. Methanol was used instead of the reagents in the blank. Quercetin was used to form a curve calibration (y = 0.0109X + 0.1081; R^2^ = 0.9979) and the total flavonol content was expressed as µg of quercetin equivalency per mg of dry extract.

### 3.6. GC-MS Analysis

GC-MS data for the EtOAc extract were acquired after trimethylsilylation with *N*,*O*-bis(trimethylsilyl)trifluoroacetamide (BSTFA) (Fluka, Buchs, Switzerland) as previously described [62]. GC-MS measurements were performed with an Agilent 6850 GC (Milan, Italy), equipped with an HP-5MS capillary column (stationary phase: 5%-phenyl-methylpolysiloxane; length: 30 m; ID: 0.25 mm; film thickness: 0.25 µm), coupled to an Agilent 5973 Inert MS detector operated in the full scan mode (*m/z* 35–550) at a frequency of 3.9 Hz and with the EI ion source and quadrupole mass filter temperatures kept, respectively, at 200 °C and 250 °C. Helium was used as the carrier gas at a flow rate of 1 mL/min. The injector temperature was 250 °C and the temperature ramp raised the column temperature from 70 °C to 280 °C: 70 °C for 1 min; 10 °C/min until 170 °C was reached; and 30 °C/min until 280 °C was reached. It was then held at 280 °C for 5 min. The solvent delay was 4 min. Metabolites were identified by comparing their EI mass spectra at 70 eV with mass spectra present in the NIST 20 mass spectral library [63]. The identification was further supported by the Kovats retention index (RI), calculated for each analyte by the Kovats equation, using the standard n-alkane mixture in the range C7–C40.

### 3.7. Biological Activities Evaluation In Vitro

#### 3.7.1. Antioxidant Ability

(a) 2,2-diphenyl-1-picrylhydrazyl (DPPH^•^) scavenging assay

With minor adjustments, the DPPH^•^ free radical scavenging assay was carried out using the procedure of Tel et al. [64]. The EtOAc sample’s various dilutions were made in Methanol. In a 96-well microplate, 160 µL of methanolic DPPH^•^ solution (0.1 mM) was mixed with 40 µL of different sample concentrations. The control was a combination of solvent and DPPH^•^ reagent. Using a microplate reader, the absorbance of each solution was measured at 517 nm following a 15 min dark incubation period at room temperature. The following formula was used to determine the percent (%) reduction of the DPPH^•^ radical:

$${DPPH}^{•}\;scavenging\;effect\left(\%\right)=\frac{A_{Control}-A_{sample}}{A_{Control}}\times 100$$

where:

Acontrol: negative control absorbance

Asample: extract (or standard) absorbance

(b) ABTS^•+^ Radical Cation Decolorization Assay

Re et al.’s [65] radical cation decolorization assay was used to measure the antiradical activity. Potassium persulfate solution (2.45 mM; 0.33 mg/mL) and ABTS^•+^ solution (7 mM; 1.92 mg/mL) were combined to create ABTS^•+^ solution. Before being used, the combination was kept at room temperature in the dark for 16 h. After diluting and adjusting, this solution had an absorbance of 0.70 ± 0.02 at 734 nm. A volume of 40 µL of the EtOAc sample at various concentrations was mixed with 160 µL of ABTS^•+^. The mixture was allowed to sit at room temperature for 10 min in the dark. Methanol and ABTS^•+^ solution served as the blank. The same DPPH^•^ formula was used to compute the inhibition %.

(c) Phenanthroline assay

The test developed by Szydłowska-Czerniak et al. [66] was used to measure the antioxidant ability. A volume of 10 µL of EtOAc fraction at different concentrations was placed in a 96-well microplate along with 50 µL of FeCl_3_ (0.2%), 30 µL of phenanthroline (0.5%), and 110 µL of methanol. Following a 20 min incubation period at 30 °C, the sample and standards were measured at 510 nm.

(d) Ferric Reducing Antioxidant Power (FRAP)

The FRAP test was carried out according to the method of Oyaizu [67]. For this, 10 µL of EtOAc extract at different concentrations were mixed with 40 µL of phosphate buffer (pH 6.6) and 50 µL of potassium ferricyanide solution. After an incubation of 20 min at 50 °C, the reaction was stopped by adding 50 µL of trichloroacetic acid (TCA). Then, 40 µL of distilled water and 10 µL of anhydrous iron chloride solution were added. Absorbance was measured at 700 nm using methanol as a blank.

Trolox and ascorbic acid were used as standards for all assays. The results were expressed as IC_50_ values (μg/mL), corresponding to the 50% inhibition concentration, in both the DPPH^•^ and ABTS^•+^ tests, and as A_0.50_ (μg/mL), corresponding to the concentration indicating 0.50 of absorbance, in the phenanthroline and FRAP assays.

#### 3.7.2. Antibacterial Activity

(a) Microorganisms

Five bacterial strains were used in this study to examine the antibacterial capacity of EtOAc extract, including two Gram-positive bacteria: *Staphylococcus aureus* (ATCC 25923) and *Bacillus subtilis* (ATCC 6633), and three Gram-negative bacteria: *Escherichia coli* (ATCC 25922), *Klebsiella pneumoniae* (ATCC 700603), and *Salmonella typhimurium* (ATCC 14028).

(b) Antibacterial effect using well diffusion method

The antibacterial assay was carried out according to a modified method from Bramki et al. [68], using a well diffusion method. Bacterial suspensions were spread on the surface of Mueller–Hinton agar plates. The plates were perforated to form wells of 6 mm in diameter. The wells were filled with EtOAc fraction (40 µL) at a concentration of 50 mg/mL in dimethyl-sulfoxide (DMSO) (2 mg in each well). The plates were incubated at 37 °C for 24 h. DMSO was used as a negative control. Gentamicin was used as a positive control (10 µg). The minimum inhibitory concentrations (MIC) were determined by testing different concentrations of the extract, ranging from 25 mg/mL to 0.39 mg/mL [69]. The antibacterial activity was determined by measuring the inhibition zones surrounding the wells (mm).

### 3.8. Statistical Analysis

All measurements were performed three times for each treatment. Statistical analyses of the data were performed using SPSS software (version 25.0). Results were analyzed by one-way analysis of variance (ANOVA) followed by Tukey HSD post hoc test for multiple comparisons. Differences were considered significant at *p* < 0.05.

### 3.9. In Silico Study

#### 3.9.1. Computational Analysis of Antibacterial Activity

To evaluate the antibacterial activity of a plant extract in EtOAc against *S. aureus*, *B. subtilis*, *E. coli*, *K. pneumoniae*, and *S. typhimurium*, a molecular docking analysis was conducted using MOE 2015.10 (Molecular Operating Environment) software. The target enzymes selected for this study included 3G75 (DNA gyrase of *S. aureus*), 3G7E (DNA gyrase of *E. coli*), 4DDQ (DNA gyrase of *B. subtilis*), 5EIX (topoisomerase of *K. pneumoniae*), and 6J90 (DNA gyrase of *S. typhimurium*).

Initially, the three-dimensional structures of the target enzymes were retrieved from the Protein Data Bank (PDB). The structures were prepared using the MOE software by adding hydrogen atoms, assigning correct bond orders, and optimizing the protonation states of the amino acids. Water molecules and other heteroatoms were removed to ensure a clean docking environment. The plant extract compounds were prepared by drawing their chemical structures using the MOE Builder tool and subsequently energy-minimized to obtain their stable conformations.

For the docking studies, the active sites of the enzymes were defined based on the co-crystallized ligands or known residues. The prepared plant extract compounds were then docked into the active sites of the enzymes using the MOE Dock tool. During the docking process, the placement methodology was set to ‘Triangle Matcher’ for pose generation, and the scoring function used was ‘London dG’ for initial scoring, followed by ‘GBVI/WSA dG’ for rescoring.

The docking results were analyzed to identify the binding poses of the compounds, their interaction energies, and the key interactions between the compounds and the active site residues of the enzymes. The best-docked poses were selected based on the highest binding affinity and the most favorable interactions. Visualization of the docking poses and interactions was performed using discovery studio software to understand the binding mechanism and to identify potential lead compounds.

#### 3.9.2. ADMET Properties

(a) Determination of canonical SMILES of molecules

The Pubchem web server was used to determine the canonical SMILES of each compound (accessed 9 July 2024), in order to predict the possible biological activities of molecules found and identified in EtOAc extract obtained from *R. ulmifolius* leaves.

(b) Physicochemical Properties

To predict the physicochemical properties of the identified molecules, Lipinski’s Rule of Five must be followed, using the online platform of Swiss ADME (http://www.swissadme.ch/, accessed on 9 July 2024) and ADMET SAR3 (http://lmmd.ecust.edu.cn/admetsar3/, accessed 5 August 2024). The parameters predicted were as follows: size, polarity, lipophilicity, insolubility, flexibility, and unsaturation. According to Lipinski’s Rule of Five, which reported that molecules suitable as a drug need to have no more than 5 hydrogen bond donors, no more than 10 hydrogen bond acceptors, and a molecular weight less than 500 Daltons. Also, a coefficient logP of no more than 5 as well as molecules presenting high bioavailability could allow a substance to be proposed as a drug likeness agent. Veber [47] set other requirements for the oral administration of a drug including flexibility and polar surface area (PSA), with the polar surface area being less than 140 A°.

(c) ADMET properties determination

The pharmacokinetic properties (absorption, distribution, metabolism, elimination) and toxicity profile of the mentioned compounds were predicted using the online platform of admetSAR 3.0 (http://lmmd.ecust.edu.cn/admetsar3/, accessed 5 August 2024), as in the protocol of Dassamiour et al. [70]. Different parameters were checked in the presence of the bioactive molecules determined, including the following: human intestinal absorption (HIA), CaCO_2_ permeability, blood–brain barrier (BBB), *p*-glycoprotein substrates and inhibitors, cytochrome P450 (CYP) classes substrates and inhibitors, OCT2, OATP1B3, OATP1B1, OATP2B1, BSEP inhibitors, skin sensitization, eye corrosion and irritation, mitochondrial and nephrotoxicity, and acute oral toxicity (AOT).

(d) Cytotoxicity of the compound’s prediction

Using the canonical SMILES of the various compounds, the cytotoxic outcomes on human cell tumors of the compounds found in EtOAc extract from *R. ulmifolius* leaves was checked and predicted using the online CLC-Pred: in silico prediction of cytotoxicity for tumor and non-tumor cell lines (http://www.way2drug.com/Cell-line/index.php, accessed on 9 July 2024) server. Two constants, Pa (Probability to be Active) and Pi (Probability to be Inactive), must be determined in order to perform this test. A molecule is only deemed active when Pa is greater than Pi.

## 4. Conclusions

This study aimed to valorize *R. ulmifolius* by assessing its phytochemical composition and evaluating its biological activities in vitro and in silico. The extraction by ethyl acetate yielded a high quantity (4.4%), reflecting the abundance of bioactive compounds present in the leaves of the plant. In addition, the results of phytochemical screening revealed the presence of tannins, flavonoids, coumarins, saponins, and anthocyanins. The quantitative estimation of polyphenols, flavonoids, and flavonols showed that the extract was rich in these metabolites. Four main compounds were identified in the organic extract by GC-MS analysis: d-(-)-fructofuranose, gallic acid, caffeic acid, and catechin. The results of antioxidant activity revealed that EtOAc extract exhibited significant radical scavenging and metal chelating effects, especially against ABTS^•+^ free radicals, with an IC_50_ value = 4.20 ± 0.13 μg/mL and phenanthroline with an A_0.5_ value of 21.22 ± 0.59 μg/mL compared to standards. Furthermore, the antibacterial activity test revealed that the extract had a significant growth inhibitory effect on all tested bacterial strains compared to gentamicin. The molecular docking analysis highlighted the binding affinities and interaction profiles of catechin, a compound from the plant extract, against key bacterial enzymes. Although catechin generally showed lower binding energies compared to the co-crystallized ligands, it formed significant hydrogen and hydrophobic interactions with crucial active site residues. This suggests that catechin and potentially other compounds in the plant extract could serve as effective antibacterial agents, warranting further investigation and optimization. The ADMET properties outcomes showed that caffeic acid was well absorbed by the BBB and the human intestine, while catechin was well absorbed in CaCO_2_. Results of 20% bioavailability (F20) and 30% bioavailability (F30) indicated that caffeic acid presented high bioavailability. All molecules can be inhibitors of OATP1B1 and OATP1B3. In the same context, catechin displayed a strong interaction with Plasma PPB, followed by gallic acid and caffeic acid. No molecules can be either a substrate or inhibitor of Pgp. Metabolically, catechin can act as an inhibitor of CYP1A2, followed by gallic acid. The outcomes stated that catechin was well eliminated by plasma clearance (CLp), while all molecules can be eliminated renally (CLr). Our results suggest that *R. ulmifolius* could be a potential source of natural antioxidants and antimicrobials in the prevention of many diseases associated with oxidative stress and infections.

## Figures and Tables

**Table 1:** Results of different phytochemicals present in *R. ulmifolius* extract.

Phytochemicals	Tannins	Flavonoids	Alkaloids	Coumarins	Sterols and Steroids	Triterpenes	Saponins	Reducing Sugars	Anthocyanins
**Results**	+++	+	−	+	−	−	+	−	+

+: Present, −: Absent.

**Table 2:** Total phenolic, total flavonoid, and flavonol contents in EtOAc fraction extracted from *R. ulmifolius* leaves (n = 3).

Total Phenolic Content (µg GAE/mg)	Flavonoid Content (µg QE/mg)	Flavonol Content (µg QE/mg)
523.25 ± 3.53	20.41 ± 1.80	9.62 ± 0.51

**Table 3:** List of compounds identified in the crude EtOAc extract. RT = retention time; RI = Kovats retention index; TMS = trimethylsilyl function, (CH_3_)_3_Si-.

Compound	RT	RI
d-(-)-Fructofuranose, 5TMS	12.669	1831
Gallic acid, 4TMS	13.28	1974
Caffeic acid, 3TMS	13.916	2148
Catechin, 5TMS	18.28	2900

**Table 4:** In vitro antioxidant activity via DPPH^•^, ABTS^•+^, phenantroline, and FRAP methods.

	IC_50_ (µg/mL)		A_0.5_ (µg/mL)	
	DPPH^•^	ABTS^•+^	Phenantroline	FRAP
**EtOAc**	98.82 ± 1.01 ^b^	4.20 ± 0.13 ^b^	21.22 ± 0.59 ^c^	94.09 ± 1.40 ^b^
**Trolox**	5.14 ± 0.09 ^a^	3.27 ± 0.17 ^a^	5.21 ± 0.07 ^b^	5.43 ± 0.31 ^a^
**Ascorbic acid**	4.40 ± 0.10 ^a^	3.07 ± 0.05 ^a^	3.08 ± 0.05 ^a^	3.76 ± 0.23 ^a^

Values with different letters are significantly different at *p* < 0.05 according to Tukey’s HSD test.

**Table 5:** Results of antibacterial activity of EtOAc extract.

	Strains Tested									
	*S. aureus*		*B. subtilis*		*E. coli*		*K. pneumoniae*		*S. typhimurium*	
	Inhibition Zone	MIC	Inhibition Zone	MIC	Inhibition Zone	MIC	Inhibition Zone	MIC	Inhibition Zone	MIC
**EtOAc**	22 ± 0.6 ^b^	0.78	24.5 ± 0.3 ^a^	0.78	21 ± 0.2 ^c^	0.78	20 ± 0.5 ^d^	3.12	20.2 ± 0.4 ^b^	0.78
**Gentamicin**	18 ± 0.5 ^f^	−	19.1 ± 0.8 ^e^	−	16.2 ± 0.9 ^g^	−	10.0 ± 0.2 ^j^	−	15.5 ± 0.7 ^h^	−

−: not determined; Inhibition zone (mm); MIC (mg/mL); Values with different letters are significantly different at *p* < 0.05 according to Tukey’s HSD test.

**Table 6:** Docking results of the reference molecule and the best-docked compound for each studied enzyme.

			Binding Energy (Kcal/mol)	Hydrogen Interactions (Distance Å)	Hydrophobic Interactions
3G7E	Co-crystallized ligand	B46	−10.7579	Asp73 (1.69), Asn56 (1.88), Arg136 (2.46), Gly102 (2.82), Gly77 (2.60), Arg76 (3.05), Asp49 (2.52)	Val43, Val120, Leu130, Val167, Met95, Ile94, His116, Ala47, Ile78, Pro79
	Best docked compound	Catechin	−5.6915	Asn46 (2.71)	Ala47, Ile78
3G75	Co-crystallized ligand	B48	−6.1928	Asp81 (1.82), Ser55 (3.39)	Ile175, Val79, Ile51, Ile86, Gly85, Asn54
	Best docked compound	Catechin	−6.1220	Gly85 (2.21), Asp81 (2.80), Asp81 (2.95)	Ale102, Ile86
5EIX	Co-crystallized ligand	LFX	−6.3059	Ser1080 (2.90), Lys444 (2.73), Lys442 (1.83)	-
	Best docked compound	Catechin	−3.6767	Glu1084 (2.63), Gly1078 (2.54)	Asp495, Ala1081, His1077
6J90	Co-crystallized ligand	ATP	−10.3302	Asp73 (1.84), Gly102 (1.90), Ser121 (2.76), Val118 (1.66), His116 (2.40), Leu115 (1.73), Gly119 (2.88), Gly117 (2.64), val120 (1.77), Asn46 (1.80)	Ile78
	Best docked compound	Catechin	−7.0767	Ans46 (2.37), Asp73 (1.96), Gly102 (2.95)	Ile78, Ile94, Val120
4DDQ	Best docked compound	Catechin	−6.3718	Ile183 (1.96), Gnl267 (2.72), Tyr99 (2.87), Arg92 (3.43), Gly41 (1.80)	Ala171, Lys43, His46, Ala172

**Table 7:** In silico cytotoxic effect prediction of compounds determined in EtOAc extract (Pa > 0.5).

Compound	Pa	Pi	Cell-Line (CL)	CL Full Name	Tissue	Tumor Type
**d** **-(-)-Fructofuranose**	0.551	0.005	CFPAC-1	Pancreatic carcinoma	Pancreas	Carcinoma
	0.520	0.004	SH-SY5Y	Bone marrow neuroblastoma	Brain	Neuroblastoma
**Gallic acid**	0.591	0.028	Hs 683	Oligodendroglioma		Glioma
**Caffeic acid**	0.607	0.010	IGROV-1	Ovarian adenocarcinoma	Ovarium	Adenocarcinoma
	0.538	0.021	K562	Erythroleukemia	Hematopoietic and lymphoid tissue	Leukemia
**Catechin**	0.556	0.006	NCI-H187	Small cell lung carcinoma	Lung	Carcinoma
	0.527	0.048	Hs 683	Oligodendroglioma	Brain	Glioma

## Data Availability

The original contributions presented in the study are included in the article/supplementary material, further inquiries can be directed to the corresponding authors.

## Supplementary Materials

The following supporting information can be downloaded at: https://www.mdpi.com/article/10.3390/plants13233425/s1, Figure S1. Annotated total ion current chromatogram (TICC) from GC-MS analysis of crude EtOAc extract of *R. ulmifolius* leaves. Table S1. Physicochemical properties of 4 compounds determined by GC-MS in EtOAc extract from *R. ulmifolius* leaves; Figure S2. Prediction of the physicochemical properties of the selected compounds; Figure S3. Prediction of absorption and brain penetration of molecules determined in ethyl acetate extract from *R. ulmifolius* Schott using Swiss ADME server; Table S2. Prediction of the ADMET properties and toxicity of the four tested compounds.
