# Supplementary material for: In Vitro and In Silico Biological Activities Investigation of Ethyl Acetate Extract of Rubus ulmifolius Schott Leaves Collected in Algeria
Source: Plants (Basel). 2024 Dec 6;13(23):3425. doi: 10.3390/plants13233425 (PMC11644080; doi:10.3390/plants13233425)
Supplement: Supplementary file 1 [file plants-13-03425-s001.zip › plants-3298006-supplementary.pdf]

SUPPLEMENTARY DATA

In vitro and *in silico* biological activities investigation of ethyl acetate extract of *Rubus ulmifolius* Schott leaves collected in Algeria

Amina Bramki [1], Djamila Benouchenne [2,3], Maria Michela Salvatore [4], Ouided Benslama [5] , Anna Andolfi [4], Noureddine Rahim [6], Mohamed Moussaoui [7], Sourore Ramoul [2], Sirine Nessah [2], Ghozlane Barboucha [6], Chawki Bensouici [8], Alessio Cimmino [4], Jesùs Garcìa Zorrilla [4,9,*] and Marco Masi [4,*]

[1] Laboratory of BioEngineering, Higher National School of Biotechnology Taoufik KHAZNADAR, nouveau Pôle universitaire Ali mendjeli, BP. E66, Constantine, 25100, Algeria; a.bramki@ensbiotech.edu.dz

[2] Higher National School of Biotechnology Taoufik KHAZNADAR, nouveau Pôle universitaire Ali mendjeli, BP. E66, Constantine, 25100, Algeria; d.benouchenne@ensbiotech.edu.dz ; s.ramoul98@gmail.com ; sirinsirin2000hh@gmail.com

[3] Laboratory of Genetic, Biochemistry and Plants Biotechnology, Faculty of Natural and Life Sciences, University of Mentouri Brothers, Constantine 1, 25000 Constantine, Algeria

[4] Department of Chemical Sciences, University of Naples Federico II, 80126 Naples, Italy; mariamichela.salvatore@unina.it ; andolfi@unina.it ; alessio.cimmino@unina.it ; marco.masi@unina.it

[5] Laboratory of Natural Substances, Biomolecules, and Biotechnological Applications, Department of Natural and Life Sciences, Larbi Ben M'Hidi University, Oum El Bouaghi, 04000, Algeria; ouided.benslama@univ-oeb.dz

[6] Biotechnologies Laboratory, Higher National School of Biotechnology Taoufik Khaznadar, Nouveau Pôle Universitaire Ali Mendjeli, BP. E66, Constantine, 25100, Algeria; n.rahim@ensbiotech.edu.dz ; g.barb@ensbiotech.edu.dz

[7] Pharmaceutical Sciences Research Center, Constantine, 25100, Algeria; mohamed.moussaoui@univ-bejaia.dz

[8] Biotechnology Research Center. UV 03, BP. E73, Ali Mendjeli, Constantine, 25016, Algeria; c.bensouici@crbt.dz

[9] Allelopathy Group, Department of Organic Chemistry, Facultad de Ciencias, Institute of Biomolecules (INBIO), University of Cadiz, 11510 Puerto Real, Spain; jesus.zorrilla@uca.es

* Correspondence: marco.masi@unina.it (M.M); jesus.zorrilla@uca.es (J. G.Z.)

Table S1 Physicochemical properties of 4 compounds determined by GC/MS in EtOAc extract from *R. ulmifolius* leaves.

Molecule	MW (g/mol)	Log (S)	N°-rotatable bonds	N°-H bond acceptors	N°-H bond donors	TPSA (A°)	Consensus Log P	
**D-(-)-Fructofuranose**	340.12	1.96	6	12	7	223.06	-3.67	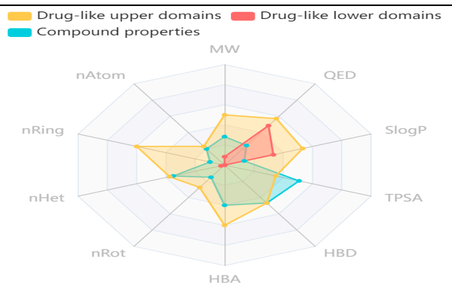
**Gallic acid**	170.12	-1.64	1	5	4	97.99	0.21	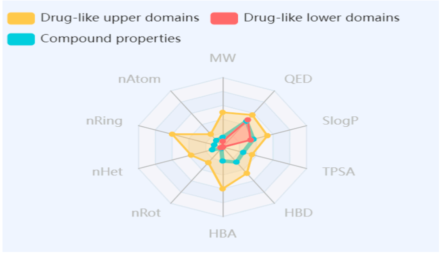
**Caffeic acid**	180.16	-1.89	2	4	3	77.76	0.93	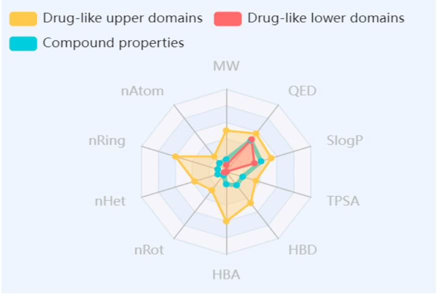
**Catechin**	290.27	-2.22	1	6	5	110.38	0.85	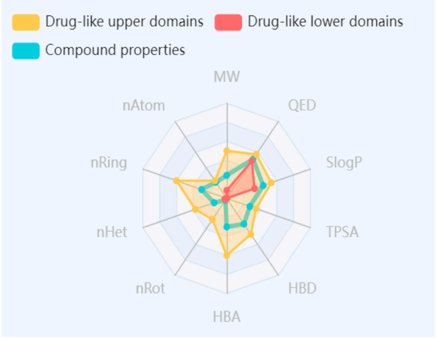

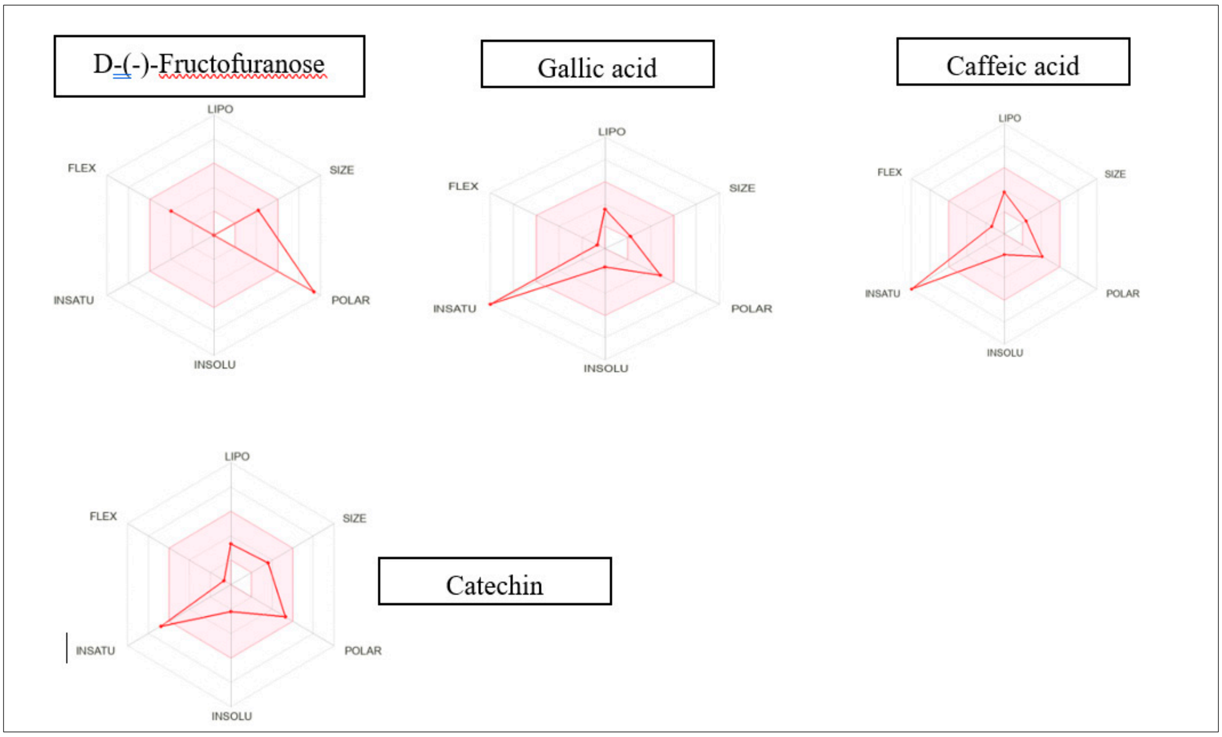

LIPO: lipophilicity, INSOLU: insolubility, INSATU: insaturation, FLEX: flexibility, POLAR: polarity

Figure S1. Prediction of the physicochemical properties of the selected compounds.

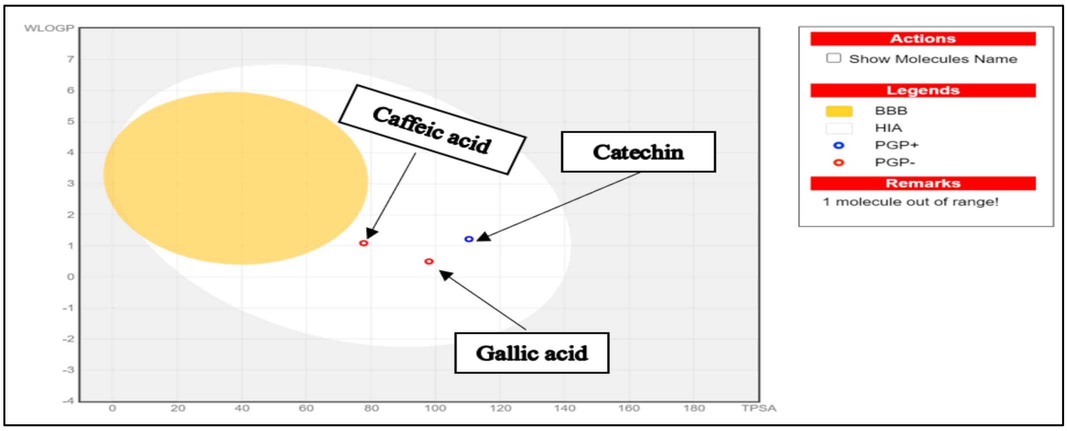

BBB: Blood-Brain Barrier; **HIA:** Human Intestinal Absorption; **PGP+**: P-glycoprotein substrate; **PGP-**: P-glycoprotein inhibitor.

Figure S2. Prediction of absorption and brain penetration of molecules determined in ethyl acetate extract from *R. ulmifolius* Schott using Swiss ADME server.

Table S2. Prediction of the ADMET properties and toxicity of the four tested compounds.

Paramentes		D-(-)-Fructofuranose		Gallic acid		Caffeic acid		Catechin	
	pKa	4.32	38.43%	1.76	20.31%	3.72	34.21%	3.09	29.75%
	Acidic pKa	3.94	25.8%	1.93	11.23%	3.58	23.2%	3.17	20.2%
	Basic pKa	6.92	64.9%	3.32	39.45%	4.19	45.63%	4.48	47.63%
	Caco-2	0	0.3%	0	5.09%	0	34.3%	-6.05	42.21%
	HIA	0	4.4%	1	53.2%	1	83.5%	1	56.49%
	MDCK	0	31.2%	0	36.3%	0	41.4%	0	35.7%
	F50%	0	7.2%	0	23.4%	0	47.5%	0	9%
	F30%	0	6.3%	0	31.8%	1	59.6%	0	15.4%
	F20%	0	8.5%	0	38.3%	1	71.7%	0	20.5%

**Distribution**	BBB	0	8.59%	0	14.19%	1	72.3%	0	14.6%
	OATP1B1 inhibitor	1	87%	1	82.7%	1	92.5%	1	83.5%
	OATP1B3 inhibitor	1	90.5%	1	82.5%	1	95.7%	1	81.1%
	OATP2B1 inhibitor	0	26.1%	0	49.7%	0	25.6%	0	44.3%
	OCT1 inhibitor	0	4.5%	0	16.2%	0	11.8%	0	18.5%
	OCT2 inhibitor	0	8.69%	0	16.1%	0	8.1%	0	24%
	BCRP inhibitor	0	5.9%	0	29.3%	0	22.2%	0	32.4%
	BSEP inhibitor	0	4.5%	0	10.7%	0	7.8%	0	20.5%
	MATE1 inhibitor	0	5.9%	0	22.9%	0	5.7%	0	25.1%
	Pgp inhibitor	0	2.7%	0	9.3%	0	6.2%	0	16.5%
	Pgp substrate	0	41.6%	0	11.1%	0	5.6%	0	30.4%
	PPB	0	28.1%	1	60.3%	1	58.5%	1	69.39%
	VDss	-0.35	27.43%	-0.65	16.67%	-0.58	18.98%	-0.27	30.36%
**Metabolism**	CYP1A2 inhibitor	0	0.9%	1	50.5%	0	16.2%	1	62.8%

	CYP3A4 inhibitor	0	0.6%	0	2.9%	0	0.6%	0	11.7%
	CYP2B6 inhibitor	0	8.59%	0	27.9%	0	14.4%	0	21.5%
	CYP2C9 inhibitor	0	0.8%	0	18.7%	0	3.2%	0	25.8%
	CYP2C19 inhibitor	0	2%	0	8.3%	0	2%	0	19.7%
	CYP2D6 inhibitor	0	3.2%	0	13.4%	0	1.8%	0	20.9%
	CYP1A2 substrate	0	0.7%	0	6.7%	0	2.6%	0	2.8%
	CYP3A4 substrate	0	2.7%	0	5%	0	3.9%	0	3.3%
	CYP2B6 substrate	0	3%	0	5%	0	8.4%	0	2.7%
	CYP2C9 substrate	0	0.7%	0	4.8%	0	4.1%	0	1.2%
	CYP2C19 substrate	0	0.4%	0	1.7%	0	1.4%	0	0.6%
	CYP2D6 substrate	0	0.7%	0	2.8%	0	1.8%	0	1.1%
	HLM	0	6.9%	0	15.6%	0	12.6%	0	23.1%
	RLM	0	6.2%	0	13.3%	0	13.2%	0	27.4%
	UGT substrate	1	75.7%	1	96.4%	**1**	95%	1	97.5%

**Excretion**	CLp	0	6.8%	1	59.9%	1	66.9%	1	73.5%
	CLr	1	52.7%	1	70.4%	1	66.2%	1	72.9%
	T1/2	-1.08	38.42%	-0.1	69.87%	0.36	84.51%	-0.07	70.8%
	MRT	-0.94	41.81%	0.04	70.8%	0.43	82.37%	0.11	73.11%
**Organ Toxicity**	Neurotoxicity	-1.92	70.63%	-1.85	74%	-2.5	44.72%	-2.14	61.03%
	DILI	0	21.4%	**0**	39.5%	1	31.7%	0	33.6%
	hERG 1uM	0	3.8%	**0**	13.1%	0	6.5%	0	12.5%
	hERG 10uM	0	21%	**0**	34.79%	0	41.9%	0	35.5%
	hERG 30uM	0	23.7%	**1**	53.1%	1	57.8%	1	65.4%
	hERG 1-10uM	0	3.7%	0	14.09%	0	7.8%	0	13.4%
	hERG 10-30uM	0	15.3%	0	43.3%	0	50.4%	1	50.6%
	Respiratory toxicity	1	70.4%	0	39.2%	0	29.6%	1	58.5%
	Nephrotoxicity	0	49.5%	0	34.59%	0	18.3%	0	32%
	Eye corrosion	0	17.39%	0	49.7%	1	53.3%	0	9.3%

	Eye irritation	0	53.5%	1	97.9%	1	98.9%	1	84.7%
	Skin corrosion	0	41.4%	1	78.2%	1	53.3%	0	35.8%
	Acute dermal toxicity	1	57.6%	1	70.1%	0	48.6%	1	55%
	Reproductive toxicity	1	58.5%	0	40.1%	0	40.2%	1	53.2%
	Mitochondrial toxicity	0	20.3%	1	74.1%	0	43.4%	1	81.29%
	Hemolytic toxicity	1	64.5%	1	55.3%	1	67.4%	1	57.5%
	Repeated dose toxicity	0	13.3%	0	25.6%	0	20%	0	23.6%
	AOT	0	2.7%	0	20%	0	26%	0	8.59%
**Cosmetic Risk Assessment**	Eye corrosion	0	17.39%	0	49.7%	1	53.3%	0	9.3%
	Eye irritation	0	37.2%	1	97.9%	1	98.9%	1	84.7%
	Skin corrosion	0	41.4%	1	78.2%	1	55.2%	0	35.8%
	Skin irritation	1	53.5%	1	84.4%	1	81.59%	1	66%

	Skin sensitisation	0	15.9%	1	51.4%	0	43.2%	0	41%
	Acute dermal toxicity	1	57.6%	1	70.1%	0	48.6%	1	55%
	Photoinduced toxicity	0	20.1%	0	50.1%	0	48.8%	0	47.6%
	Phototoxicity	0	29.9%	0	25.8%	0	14.09%	0	25.6%
	Photoallergy	0	15.5%	0	49.3%	0	45%	0	43.8%
	Compound properties	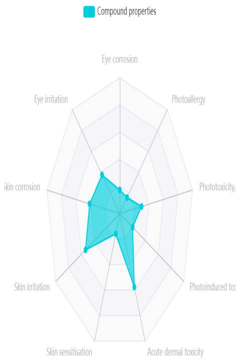		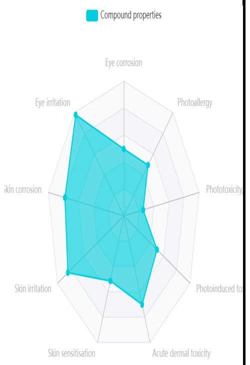		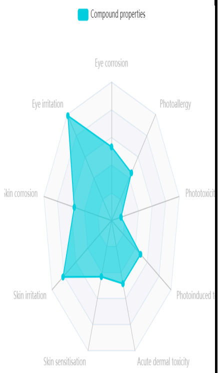		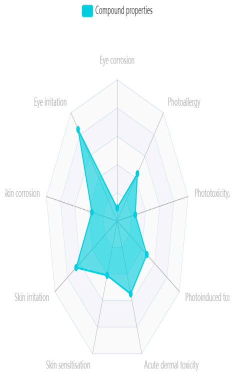	
**Medicinal Chemistry**	QED	0.24	20.47%	0.46	46.42%	0.47	47.85%	0.51	52.38%
	Lipinski Rule	Accept	100%	Accept	100%	Accept	100%	Accept	100%
	Pfizer Rule	Accept	100%	Accept	100%	Accept	100%	Accept	100%

	GSK Rule	Accept	100%	Accept	100%	Accept	100%	Accept	100%

HIA: Human Intestinal Absorption; **BBB:** Blood Brain Barrier; **F 50%, F 30%, and F 20%**; **PPB:** Plasma protein binding; **AOT:** Acute Oral Toxicity; **UGT catalyzed**: UDP-glucuronosyltransferase; **CYP:** Cytochrome isoforms; **CNp:** Plasma clearance; **CLr**: Renal clearance.
